# Risk and Protective Factors for COVID-19 Infection among Pregnant Women with Sickle Cell Trait

**DOI:** 10.1155/2024/1595091

**Published:** 2024-06-12

**Authors:** Kim Abbegail Tan Aldecoa, Camelia Arsene, Geetha Krishnamoorthy, Tiffany Chng, Garrett Cherry, Nabila Chowdhury, Ryan Clark, Dana Deeb, Lisa Deptula, Grey Dietz, Ewomamobuho Eto, Victoria Golston, Landon Lawson, Chioma Mbionwu, Obiefuna Okponyia, Jennifer Orejuela, Thomaidha Qipo, Sumit Raut, Judie Goodman

**Affiliations:** ^1^Department of Internal Medicine, Trinity Health Oakland, Pontiac, MI, USA; ^2^Wayne State University, Detroit, MI, USA; ^3^Ross University School of Medicine, Bridgetown, Barbados; ^4^Detroit Wayne County Authority Health, Detroit, MI, USA; ^5^Department of Hematology and Oncology, Trinity Health Oakland, Pontiac, MI, USA

## Abstract

Pregnant women and individuals with sickle cell trait (SCT) and underlying comorbidities are both independently more vulnerable to severe illness from coronavirus disease 2019 (COVID-19) compared to nonpregnant women and those without SCT. However, our understanding of the specific factors influencing susceptibility to COVID-19 infection among pregnant women with SCT is currently constrained by limited available data. This study aims to determine the risk and protective factors that influence the likelihood of COVID-19 infection in this population. A retrospective analysis was done among 151 women with SCT in the reproductive age group. Multivariable analysis was performed to determine the various factors affecting COVID-19 infection among pregnant women with SCT. The study found that COVID-19-vaccinated pregnant women with SCT had a 90% lower risk of contracting COVID-19 and were 9 times more likely to have a COVID-19 infection if they had a history of pulmonary conditions such as asthma or chronic obstructive pulmonary disease. The present study further emphasizes the importance of the COVID-19 vaccine in preventing infection and safeguarding the health of pregnant women with SCT, particularly those with underlying comorbidities.

## 1. Introduction

Sickle cell trait (SCT) affects almost 300 million individuals worldwide [1], with a prevalence rate of 9% among the Black population and 0.2% among Caucasians in the United States [2]. It is an autosomal recessive genetic disorder that occurs when an individual carries one abnormal hemoglobin beta gene allele (Sickle hemoglobin, HbS) and one normal beta gene allele (Adult hemoglobin, HbA). While it is commonly perceived to be a benign condition, those with SCT may develop sickle-related complications under certain conditions that increase oxygen demands [3, 4], such as COVID-19 infection causing severe pneumonia. Additional triggers include extreme conditions such as high altitude, severe dehydration, or very high-intensity physical activity [4]. Furthermore, recent evidence suggests that hemoglobin, particularly its beta subunit which is abnormal in the HbS allele, participates in innate immunity by regulating genes involved in viral recognition and mobilizing interferon response that is important for defending against viruses [5].

The incidence of COVID-19 infection in pregnant women was 3.02 times greater than that in the general population [6]. Pregnancy causes several physiological changes and alters the immune system and its response to viral infection [7, 8], which might increase the risk of any infection [9]. This includes COVID-19, which could lead to more severe symptoms, especially in the third trimester [8, 9]. The presence of certain comorbidities further worsens the severity of COVID-19 disease [10], but there is a paucity of studies available for pregnant individuals with SCT.

Pregnancy and SCT are both independently associated with more adverse COVID-19 outcomes [3, 4, 6–8]. However, the impact of the pandemic and factors affecting susceptibility to COVID-19 among pregnant women with SCT are unknown. This study aims to examine various factors that can alter the likelihood of COVID-19 among pregnant women with SCT, including vaccination against COVID-19 and influenza and the presence of underlying comorbidities such as asthma, chronic obstructive pulmonary disease (COPD), heart conditions, diabetes, and obesity. In addition, the study aims to provide insights into the fetal outcomes among pregnant women with SCT who have contracted COVID-19.

## 2. Methods

Individuals with SCT who had emergency and inpatient encounters at three teaching hospitals in Michigan from January 1, 2022, to December 31, 2022, were identified by International Classification of Diseases, Tenth Version (ICD-10) codes. Patients who had any instance of sickle cell disease in their medical records were excluded. The classification of COVID-19 vaccination status into fully, partially, or nonvaccinated categories was established according to the definitions provided by the Centers for Disease Control and Prevention (CDC) [11]. Disease severity was assessed by referencing the COVID-19 guidelines outlined on the official website of the National Institutes of Health [12]. The study protocol was approved by the Institutional Review Board at Trinity Health System in Michigan.

A total of 295 individuals with a diagnosis of SCT were screened in this retrospective chart review study. Among them, 151 women with SCT met the inclusion criteria and were within the reproductive age group range of 15–49 years [13]. Demographics, underlying comorbidities, fetal outcomes, and other clinical information were collected by Electronic Medical Records review. COVID-19 vaccination and influenza vaccination were verified from the Immunization Records Registry in Michigan. Descriptive statistical analyses were performed. Continuous variables with their mean and standard deviation and categorical variables with frequencies and percentages were presented. For categorical variables, group differences were analyzed using the Chi-square or Fisher's exact test. Differences between groups for continuous variables were measured with Student's *T*-test. Multivariable regression analysis was used to analyze the factors associated with COVID-19 infection. Statistical analyses were performed using the IBM SPSS version 29 statistical software, and a *p* value<0.05 was considered statistically significant.

## 3. Results

A total of 151 women with SCT were included in the analysis, with 34% being pregnant and 66% being nonpregnant. Approximately 85% of the participants were Black, 2.7% Caucasian, 2.0% Hispanic, 1.3% Asian. The mean age of the participants was 32 years, with pregnant individuals relatively younger compared to nonpregnant individuals with SCT (29 vs. 34 years, *p* < 0.05).  Table 1 presents an overview of the baseline demographics of the study population.

In this study, the prevalence of COVID-19 among pregnant women was 35%, compared to 31% among nonpregnant women, with no statistically significant difference (Table 2, *p* > 0.05). Most cases in both groups exhibited asymptomatic to moderate disease, with only one patient developing severe illness and no critical cases reported. Additionally, there was no mortality recorded. Table 2 provides a summary of these findings.

Among women in the reproductive age group with SCT, common symptoms reported at the time of COVID-19 diagnosis included cough (36.7%), myalgia (16.3%), and shortness of breath (12.2%). Additionally, 12.2% were incidentally found to have COVID-19 infection or were asymptomatic. In pregnant women, the most frequently reported chief complaints included cough (27.8%), congestion (27.8%), and sore throat (22.2%), as outlined in Table 3. Some patients reported multiple chief complaints, which were documented accordingly in the same table.

The study conducted a multivariable regression analysis and found that COVID-19-vaccinated pregnant women with SCT had a 90% lower risk of contracting COVID-19 infection (OR = 0.102 with 95% CI: 0.011-0.950). The study also found that pregnant women were 9 times more likely to have a COVID-19 infection if they have a history of pulmonary conditions such as asthma or chronic obstructive pulmonary disease (OR = 9.375 with 95% CI: 1.090-80.627). There were no other protective or risk factors identified that showed a significant statistical association with COVID-19 infection among pregnant women in this study, as presented in Table 4.

Most pregnant women with SCT who contracted COVID-19 had mild-moderate disease, except for one patient who developed severe disease in the third trimester, warranting premature delivery of an infant (34 weeks, low birth weight) via emergency caesarean section. Overall, 72.2% did not have fetal complications, except for 3 newborns who were delivered preterm (<37 weeks) and 2 with low birth weight (<2500 g). While 2 newborns initially had a low Apgar score (APGAR<7) at 1 minute, their condition eventually improved after 5 minutes. Overall, all newborns delivered to COVID-19-infected women with SCT had a good 5-minute Apgar score.  Table 5 provides a summary of the outcome of COVID-19 infection among women with SCT.

## 4. Discussion

The study reveals the effectiveness of the COVID-19 vaccine in preventing infection among pregnant women with SCT. It also provides the most prevalent manifestations of COVID-19 infection among pregnant women and nonpregnant women of reproductive age. Furthermore, it provides insights into the fetal outcomes of pregnant women with SCT who have contracted COVID-19.

The COVID-19 vaccination rate within our cohort was relatively low at 35%. A concurrent study conducted by the CDC within the same time period reported higher vaccination rates among pregnant women in the general population, irrespective of SCT status: 64.9% had received at least one dose of the COVID-19 vaccine, 58.7% completed the primary vaccination series, and 27.3% received the booster dose [14]. Understanding the underlying reasons for this hesitancy is crucial. Research indicates that Black individuals, comprising the majority of our sample population and representing a prominent demographic with SCT [2], exhibit lower rates of vaccine acceptance compared to other demographics [15, 16]. Additional factors contributing to COVID-19 vaccination hesitancy during pregnancy include concerns about vaccine side effects, perceptions of vaccines as being too new, and government mistrust [17]. Despite these concerns, leading medical societies, including the American College of Obstetricians and Gynecologists (ACOG), have universally recommended COVID-19 vaccination for pregnant individuals in any trimester [18]. Furthermore, studies conducted by the CDC have not identified any safety concerns associated with COVID-19 vaccination in pregnant individuals, with reported side effects comparable to those experienced by nonpregnant individuals [18, 19].

COVID-19 vaccination during pregnancy has been well documented as safe for newborns and provides protection against adverse outcomes [19, 20]. Pregnant women who receive the vaccine generate virus-specific antibodies for SARS-CoV-2 and its variants, including Delta and Omicron. These antibodies are transmitted to their newborns through the placenta, offering protection during the vulnerable period after birth [20]. Moreover, both maternal and cord blood exhibit significantly higher levels of SARS-CoV-2 antibodies after receiving a booster dose, suggesting enhanced immunity in newborns against COVID-19 [20]. Future strategies are recommended to increase vaccine confidence and coverage among individuals with SCT, especially those in the reproductive age group, with a particular emphasis on high-risk pregnant women.

Our study revealed that among pregnant women with SCT, the most prevalent symptoms at the time of COVID-19 diagnosis were cough (27.8%), congestion (27.8%), and sore throat (22.2%). In contrast, a systematic review of pregnant women in the general population highlighted cough, fever, and myalgia as the most commonly reported symptoms, with sore throat (7^th^) and nasal congestion (17^th^) occurring less frequently [21]. In our study, fever (2.0%) and myalgia (2.0%) were among the least reported symptoms.

The study revealed that pregnant women with SCT who have a history of asthma or COPD are at a heightened risk of contracting COVID-19. This aligns with recent findings from the CDC, which identified underlying comorbidities, including asthma and COPD, as significant risk factors for COVID-19 infection and hospitalization in the general population [10, 22, 23]. Angiotensin-converting enzyme 2 (ACE2) might have played a role. SARS-CoV-2 utilizes ACE2 as a receptor to infiltrate cells, with higher levels of this enzyme found in the respiratory tract epithelial cells, particularly in individuals with COPD [24]. In asthma, there were variations in ACE2 expression due to different asthma endotypes which can influence COVID-19 susceptibility, with IL-17 potentially upregulating ACE2 expression, while IL-4 and IL-13 may downregulate it [25, 26]. Research indicates that COPD patients are at a higher risk of severe COVID-19 compared to asthmatic individuals, possibly due to the increased number of ACE2-positive cells in alveolar cells among COPD patients [26]. Furthermore, the overlapping respiratory symptoms among COVID-19, asthma, and COPD, combined with their impact on both upper and lower airways, can complicate diagnosis and management [27]. Asthma symptoms also often worsen during pregnancy [28], and COVID-19 infection can trigger asthma attacks [29]. Pregnancy-related physiological changes, such as uterine distension and hormonal fluctuations, can exacerbate respiratory issues by affecting lung volumes (reduction of functional residual capacity and expiratory reserve volume) and airway clearance, making pregnant individuals more susceptible to respiratory infections [28, 30].

The impact of SCT on pregnancy and childbirth outcomes remains relatively unexplored, with scant literature addressing maternal and neonatal outcomes among pregnant women with SCT. Despite the limited sample size in our study, we sought to shed light on these outcomes. Pregnant women, especially in the third trimester, face an elevated risk of severe illness from COVID-19 infection [8, 31], with pneumonia from various infectious sources constituting a significant cause of morbidity and mortality during pregnancy [32]. These risks are partly attributed to alterations in cell-mediated immunity [33] and the body's response to viral infections [7, 8]. Pregnancy-induced changes in immune function, characterized by a systemic shift toward T-helper type II (Th2) dominance [33], may contribute to maternal tolerance of the fetus but also heighten susceptibility to infectious diseases [33, 34]. While the impact of COVID-19 infection on pregnancy outcomes, particularly among SCT, remains inconclusive, our study revealed three preterm births (one associated with severe COVID-19 disease, with no reported fetal deaths). Other studies have shown that the severity of COVID-19 disease appears to correlate with adverse pregnancy outcomes, with moderate or higher disease severity linked to preterm birth and Neonatal Intensive Care Unit (NICU) admission [35, 36]. Conversely, asymptomatic or mild COVID-19 cases showed outcomes similar to those without SARS-CoV-2 infection [37]. Conflicting findings exist regarding fetal outcomes, with earlier studies reporting no association between COVID-19 infection and adverse neonatal outcomes [36], while recent reports suggest an increased risk of stillbirth among pregnant women with COVID-19 [38]. Our study provides valuable insights, but the limited sample size hinders our ability to draw definitive conclusions regarding the influence of SCT on pregnancy outcomes. At present, there is a dearth of studies comparing outcomes between pregnant women with and without SCT, underscoring the need for further investigation in this field.

In this study, no statistically significant association was found among pregnant women with SCT between COVID-19 infection and various factors including influenza vaccination, obesity, heart conditions, diabetes mellitus, renal disorder, stroke, transient ischemic attack, or mental health conditions. Additional studies with larger sample sizes are warranted to confirm the above findings among pregnant women with SCT. According to a recent report by the Centers for Disease Control and Prevention in the general population, heart conditions (such as heart failure, coronary artery disease, and cardiomyopathy), diabetes mellitus, cerebrovascular disease, obesity, and mental health conditions (mood disorders and schizophrenia spectrum disorders) were conclusive to have a higher risk for severe illness from COVID-19 infection [10]. Consistent with our findings, the CDC has stated that the influenza vaccine does not provide protection against COVID-19 infection [39].

Our study has some limitations that warrant consideration. Firstly, its retrospective nature restricts the ability to establish causality, emphasizing the need for prospective studies to corroborate our findings. Secondly, the relatively small sample size underscores the importance of large-scale investigations to derive more definitive conclusions. Furthermore, the limited number of individuals with severe COVID-19 disease in our study precluded a comprehensive analysis of factors associated with severe illness among this population. Future research endeavors with expanded sample sizes and prospective designs are warranted to address these limitations and provide more robust insights into the factors influencing disease outcomes and severity in pregnant women with SCT.

## 5. Summary/Conclusion

The study further highlights the importance of encouraging COVID-19 vaccination among individuals with SCT, particularly pregnant, reproductive-age women and those with underlying comorbidities. Pregnant women with SCT and underlying comorbidities should be carefully monitored to prevent severe disease, as they may be more vulnerable to COVID-19 infection. Further studies with larger sample sizes are recommended to investigate factors associated with severe COVID-19 infection among these populations.

## Figures and Tables

**Table 1:** Baseline characteristics of women with sickle cell trait (SCT) included in the study.

Baseline characteristics	Pregnant (*N* = 51)	Nonpregnant (*N* = 100)	Total (*N* = 151)
Age (Mean ± standard deviation)	29 years ±5.9	34 years ±8.6	32 years
Race			
Black	45 (88.2%)	84 (84.0%)	129 (85.4%)
Caucasian	2 (3.9%)	2 (2.0%)	4 (2.7%)
Hispanic	1 (2.0%)	2 (2.0%)	3 (2.0%)
Asian	1 (2.0%)	1 (1.0%)	2 (1.3%)
Unknown	2 (3.9%)	11 (11.0%)	13 (8.6%)
Heart conditions (such as heart failure, coronary artery disease, cardiomyopathy)	19 (37.3%)	29 (29.0%)	48 (31.8%)
Diabetes mellitus	7 (13.7%)	14 (14.0%)	21 (13.9%)
Pulmonary disease history (asthma, chronic obstructive pulmonary disease)	9 (17.6%)	28 (28.0%)	37 (24.5%)
Stroke	0 (0%)	3 (3.0%)	3 (2.0%)
Renal disease	1 (2.0%)	3 (3.0%)	4 (2.7%)
Mental health condition	13 (25.5%)	31 (31%)	44 (29.1%)
Influenza infection	3 (5.9%)	3 (3.0%)	6 (4.0%)
Influenza vaccination	23 (45.0%)	40 (40.0%)	63 (41.7%)
COVID-19 vaccination status			
(1) Vaccinated	18 (35.3%)	50 (50.0%)	68 (45.0%)
(a) Partial	5 (27.8%)	9 (18.0%)	14 (20.6%)
(b) Full	8 (44.4%)	32 (64.0%)	40 (58.8%)
(c) Booster	5 (27.8%)	9 (18.0%)	14 (20.6%)
(2) Nonvaccinated	33 (64.7%)	50 (50.0%)	83 (55.0%)

**Table 2:** Overview of COVID-19 infection among patients included in the study.

	Pregnant (*N* = 51)	Nonpregnant (*N* = 100)	*p* value
Positive for COVID-19 infection	18 (35.3%)	31 (31.0%)	0.594
(a) Asymptomatic	0 (0%)	6 (19.4%)	
(b) Mild	15 (83.3%)	16 (51.6%)	
(c) Moderate	2 (11.1%)	9 (29.0%)	
(d) Severe	1 (5.6%)	0 (0%)	
(e) Critical	0 (0%)	0 (0%)	

**Table 3:** Clinical presentation during COVID-19 diagnosis among women with SCT in the reproductive age group, stratified by pregnancy status.

Clinical presentation/chief complaints	Pregnant (*N* = 18)	Nonpregnant (*N* = 31)	Total (*N* = 49)
Asymptomatic	0 (0%)	6 (19.4%)	6 (12.2%)
Cough	5 (27.8%)	13 (41.9%)	18 (36.7%)
Congestion	5 (27.8%)	0 (0%)	5 (10.2%)
Sore throat	4 (22.2%)	1 (3.2%)	5 (10.2%)
Headache	3 (16.7%)	2 (6.5%)	5 (10.2%)
Fatigue	1 (5.6%)	2 (6.5%)	3 (6.1%)
Myalgia	2 (11.1%)	6 (19.4%)	8 (16.3%)
Fever/Chills	1 (5.6%)	3 (9.7%)	4 (8.2%)
Shortness of breath	1 (5.6%)	5 (16.1%)	6 (12.2%)
Nausea/vomiting	0 (0%)	1 (3.2%)	1 (2.0%)
Chest pain	0 (0%)	3 (9.7%)	3 (6.1%)
Altered mental status	1 (5.6%)	0 (0%)	1 (2.0%)
Ageusia/anosmia	1 (5.6%)	1 (3.2%)	2 (4.1%)
Dizziness	1 (5.6%)	1 (3.2%)	2 (4.1%)

**Table 4:** Multivariable analysis of factors affecting COVID-19 infection among pregnant women with SCT.

Factors influencing COVID-19 infection	Odds ratio	*p* value	95% CI
Age	1.020	0.755	0.901, 1.155
COVID-19 vaccination	**0.102**	**0.045**	**0.011, 0.950**
Influenza vaccination	6.398	0.066	0.883, 46.355
Obesity	0.639	0.571	0.135, 3.012
Heart conditions	2.233	0.313	0.470, 10.613
Pulmonary disease history	**9.375**	**0.041**	**1.090, 80.627**
Diabetes mellitus	0.464	0.455	0.062, 3.481
Mental health condition	0.732	0.742	0.114, 4.715

Values in bold indicate statistical significance with a *p* value less than 0.05.

**Table 5:** Summary of fetal outcome among women with SCT who contracted COVID-19 infection during pregnancy.

Trimester at the time of COVID-19 infection	COVID-19 severity	Mode of delivery	Age of gestation of delivery	APGAR score	Weight of newborn (grams)
Third	Moderate	Vaginal	≥37 weeks	Normal	Normal
First	Mild	Vaginal	≥37 weeks	Normal	Normal
Third	Severe	Caesarean	**34 weeks**	**6,9**	**2010**
Second	Mild	Caesarean	≥37 weeks	Normal	Normal
First	Mild	Caesarean	≥37 weeks	Normal	Normal
Third	Moderate	Vaginal	≥37 weeks	Normal	Normal
Second	Mild	Caesarean	**33 weeks**	**5,8**	**1980**
Third	Mild	Caesarean	≥37 weeks	Normal	Normal
Third	Mild	Caesarean	Unknown	Unknown	Unknown
First	Mild	Caesarean	**36 weeks**	Normal	Normal
Third	Mild	Vaginal	≥37 weeks	Normal	Normal
First	Mild	Vaginal	Unknown	Unknown	Unknown
Second	Mild	Caesarean	≥37 weeks	Normal	Normal
Third	Mild	Vaginal	≥37 weeks	Normal	Normal
Third	Mild	Caesarean	≥37 weeks	Normal	Normal
Third	Mild	Caesarean	≥37 weeks	Normal	Normal
Third	Mild	Caesarean	≥37 weeks	Normal	Normal
First	Mild	Vaginal	≥37 weeks	Normal	Normal

Values highlighted in bold indicate significant findings. ^*∗*^APGAR: appearance, pulse, grimace, activity, and respiration; normal = 7–10.

## Data Availability

The datasets used and/or analyzed during the study are available upon reasonable request.

## Abbreviations

SCT: Sickle cell trait
COVID-19: Coronavirus disease 2019
SARS-CoV-2: Severe acute respiratory syndrome coronavirus 2.
